# From Waste to Binder: Alkali Activation of Blended Brick and Metakaolin Residues for Design of Circular Construction Materials

**DOI:** 10.3390/polym17202720

**Published:** 2025-10-10

**Authors:** Martin Mildner, Petr Hotěk, Martina Záleská, Robert Černý, Jan Fořt

**Affiliations:** Department of Materials Engineering and Chemistry, Faculty of Civil Engineering, Czech Technical University in Prague, Thákurova 7, 166 29 Prague, Czech Republic; martin.mildner@fsv.cvut.cz (M.M.); petr.hotek@fsv.cvut.cz (P.H.); martina.zaleska@fsv.cvut.cz (M.Z.); cernyr@fsv.cvut.cz (R.Č.)

**Keywords:** alkali-activated material, brick powder, circularity, carbon footprint, blended precursor

## Abstract

Alkali-activated materials (AAMs) offer a promising low-carbon alternative to Portland cement, but their development has been dominated by fly ash and slag, whose availability is increasingly limited. This research explores waste brick powder (WBP) and metakaolin residue (RN), two abundant yet underutilized by-products, as blended precursors for sustainable binder design. The novelty lies in demonstrating how complementary chemistry between crystalline-rich WBP and amorphous RN can overcome the drawbacks of single-precursor systems while valorizing construction and industrial residues. Pastes were prepared with varying WBP/RN ratios, activated with alkaline solutions, and characterized by Vicat setting tests, isothermal calorimetry, XRD with Rietveld refinement, MIP, SEM, and mechanical testing. Carbon footprint analysis was performed to evaluate environmental performance. Results show that WBP reacts very rapidly, causing flash setting and limited long-term strength, whereas the incorporation of 30–50% RN extends setting times, sustains dissolution, and increases amorphous gel formation. These changes refine the formed reaction products, leading to compressive strengths up to 39 MPa and flexural strengths of 8 MPa at 90 days. The carbon footprint of all blends remained 392–408 kg CO_2_e/m^3^, thus providing about a 60% improvement compared to conventional Portland cement paste. The study establishes clear design rules for waste-derived blended precursors and highlights their potential as circular, low-carbon binders.

## 1. Introduction

Concrete has become the backbone of modern infrastructure, but its key component, Portland cement, represents a material paradox. It is indispensable for construction yet responsible for an estimated 7–8% of global anthropogenic CO_2_ emissions, with every ton of clinker emitting close to 0.9 t of CO_2_ from both fuel combustion and the decarbonation of limestone. This dependence on ordinary Portland cement (OPC) has persisted for more than 150 years, despite growing evidence that the process is fundamentally incompatible with the EU’s climate neutrality target for 2050 [1,2,3]. Incremental efficiency improvements such as alternative fuels, clinker substitution, and even carbon capture technologies cannot by themselves reconcile the sector’s emissions with the carbon goals given by the Paris Agreement [4]. The sector therefore requires disruptive approaches that can break its reliance on conventional clinker chemistry and deliver low-carbon binders with significantly reduced environmental footprints [5].

Alkali-activated materials (AAMs) are one of the very promising candidates for such a transformation. These binders differ fundamentally from OPC. Instead of high-temperature clinkering, they rely on the dissolution and reprecipitation of aluminosilicates in alkaline environments [6,7]. The reaction yields three-dimensional polymeric gels, typically sodium aluminosilicate hydrate (N-A-S-H) in low-calcium systems and calcium aluminosilicate hydrate (C-A-S-H) or hybrid (N,C)-A-S-H gels in higher-calcium systems [8]. AAMs are capable of delivering compressive strengths and durability comparable to or exceeding OPC, while environmental assessments consistently report reductions in CO_2_ footprint [9]. For several decades, most scientific studies on AAMs have focused on fly ash and blast furnace slag, two by-products that were available in large volumes and carried a low environmental burden when considered as wastes. However, the supply of fly ash in Europe is collapsing as coal-fired power plants are phased out under the energy transition. Concurrently, the shift from blast furnaces to electric arc furnaces in steelmaking is reducing the output of granulated slag [10,11]. This creates a paradox where AAMs have been promoted as binders of the future, but their most studied precursors are themselves materials of the past. This scarcity problem motivates the exploration of alternative aluminosilicate sources that are not tied to declining industries but instead come from abundant and continuously generated waste streams [12,13,14]. Besides FA and GBFS, metakaolin was extensively studied as a suitable precursor for alkaline activation [14]. Alkali-activated metakaolin pastes often achieve compressive strengths of 40–70 MPa, along with excellent resistance to chemical attack [15]. However, from an environmental and economic perspective, producing commercial metakaolin from high-purity clays is energy-intensive and costly [16]. However, the industrial processing of kaolin also generates significant amounts of off-spec metakaolin residues. These residues retain high reactivity but are unsuitable for premium applications such as specialty cements or paper coatings and are often landfilled or downcycled. Valorizing such residues as alkali-activation precursors represents both a resource efficiency strategy and a pathway for industrial symbiosis [17].

Among the most promising of these alternative resources are construction and demolition waste (CDW) fines. The European Union generates more than 350 million tons of CDW each year, nearly one-third of total waste production [18]. Much of this material is already recycled as aggregate, yet the fine fractions, typically below 2 mm, remain underutilized. These fines accumulate in recycling plants without any relevant reuse scenario, where they pose a logistical burden. Waste brick powder (WBP), originating from demolition of masonry and from defective products rejected during ceramic production, poses a very abundant material originating in the building industry [19,20]. However, the main challenge associated with WBP is based on limited reactivity. Mineralogically, fired brick fines are rich in crystalline phases such as quartz, feldspar or anorthite, and mullite, while their amorphous content is generally only 15–25%, depending on firing temperature and feedstock composition [20,21]. This low amorphous fraction constrains dissolution of silicon and aluminum species during alkaline activation, limiting gel formation and long-term strength development, as reported in the relevant scientific studies. Shen et al. [22] reported that alkali-activated brick-powder pastes exhibited very fast early hardening, with compressive strengths of up to about 31 MPa under accelerated curing, but reactivity was strongly dependent on activator dosage and particle fineness. Sharmin et al. [23] demonstrated that partial replacement of fly ash by waste clay brick powder in AAM mortars significantly altered pore structure and strength, with optimized blends achieving competitive mechanical performance. However, pure WBP binders do not provide sufficient performance. Cardoza and Colorado [24] similarly observed that activating red brick waste with partial OPC addition improved both workability and early strength, but brick-only binders were insufficient.

Blended waste brick powder with more reactive precursors offers a rational strategy to exploit the complementary strengths of each material while compensating for their individual weaknesses [25]. Especially for those with low initial setting, where waste brick powder contributes Ca-bearing crystalline phases that promote early stiffening, while a more reactive precursor provides a reservoir of amorphous aluminosilicates that sustain dissolution and gel formation over time. Statkauskas et al. [26] investigated ceramic brick waste blended with metakaolin waste and phosphogypsum and observed that increasing the metakaolin fraction improved strength development and microstructural cohesion, even as total porosity increased. Alghamdi et al. [27] showed that moderate substitution of metakaolin/slag mortars with waste brick powder enhanced compressive strength by up to 30% compared to binary systems. The study confirmed that brick-derived aluminosilicates can beneficially participate when combined with more reactive phases. Jin et al. [28] further demonstrated that adding brick powder into slag geopolymers modified calorimetric profiles and refined gel structures when carefully dosed. These examples indicate that combining brick fines with reactive metakaolin residues can deliver mechanical performance exceeding that of brick-only AAMs.

Environmental implications represent an important motivation for the design of new binders, AAMs in particular. Valente et al. [29] compared AAMs and conventional cementitious binders using experimental data and LCA and showed that AAMs consistently achieved substantial reductions in global warming potential, often exceeding 40%, while achieving competitive strengths. Kravchenko et al. [30] examined AAMs produced from CDW-derived precursors and concluded that mortar-scale use of CDW precursors is environmentally preferable to concrete-scale applications for several impact categories, with benefits linked to avoided landfill impacts. Alkhawaldeh et al. [31] reported at least 35% lower CO_2_ emissions compared to conventional ceramic products, primarily due to avoided landfill and shorter logistics chains. The European Green Deal and the revised Waste Framework Directive require that at least 70% of CDW be recovered by 2035, with an emphasis on high-value rather than low-grade applications [11]. The European Environment Agency has warned that high recovery rates reported by Member States often reflect backfilling rather than true recycling [32]. Positioning waste brick powder as a binder precursor directly addresses this policy gap, moving fines into structural materials where they can substitute virgin resources and deliver clear CO_2_ savings.

Despite the promising perspectives, knowledge gaps remain; thus, systematic work is needed to establish a robust knowledge base for the design of novel AAMs without using FA or slag [33,34], but with improved mechanical strength [35]. In this regard, there is a need for more fundamental work at the paste level to clarify how different precursor combinations interact, how amorphous and crystalline fractions contribute to reaction kinetics, and how these interactions shape gel chemistry, pore structure, and ultimately, material performance.

This work addresses the dual challenge of advancing sustainable binder technologies and valorizing abundant waste streams by combining a widely available but low-reactive precursor (waste brick powder—WBP) with a reactive industrial residue from metakaolin production (RN) to form a viable alkali-activated binder. The results assess the potential of both waste sources and highlight the potential role of the WBP as the setting moderator agent. This effect is particularly beneficial when combined with activators that otherwise display excessively slow setting behavior. In contrast, when blended with RN, the system benefits from higher contents of reactive Si and Al, which promote the formation of stable (N,C)-A-S-H gels and contribute to sustained strength development. Such synergy broadens the applicability of slower activators in practical construction scenarios, where both processing window and mechanical performance are essential. The findings broaden the scope for utilizing diverse waste materials and thereby enhance material circularity in the construction industry. At the same time, they demonstrate viable pathways for substituting conventional precursors.

## 2. Materials and Methods

### 2.1. Used Materials

In designing the mix formulations, waste brick powder (WBP) originating from a construction and demolition waste recycling facility in Czechia was employed as one of the precursors. To increase its suitability for alkaline activation, the material was mechanically processed. In this regard, a prolonged milling was applied to reduce particle size and disrupt residual crystalline aggregates, thereby enhancing the available surface area for dissolution. To minimize the influence of inert coarse particles, grains exceeding 0.125 mm were separated by sieving, and only the finer fraction was used for mix design. The treated material was subsequently dried to stabilize its condition before being combined with metakaolin residue (RN), an industrial by-product supplied by CLUZ (Czechia).

**Figure 1:**
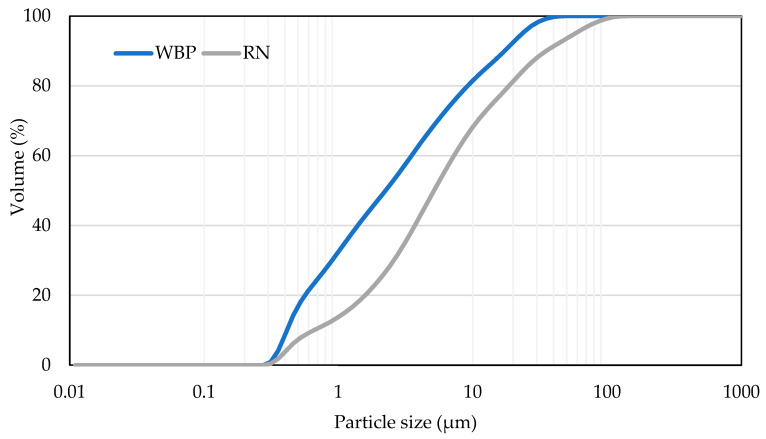
Cumulative particle size distribution of WBP and RN.

The granulometric characteristics of the processed powders were analyzed by laser diffraction, enabling precise evaluation of particle size distribution up to 2 mm. This measurement confirmed that both precursors exhibit fine fractions suitable for alkali activation, with median particle sizes (d50) of 5.14 µm for RN and 2.24 µm for WBP, indicating that WBP provides a slightly finer reactive surface (see Figure 1).

**Figure 2:**
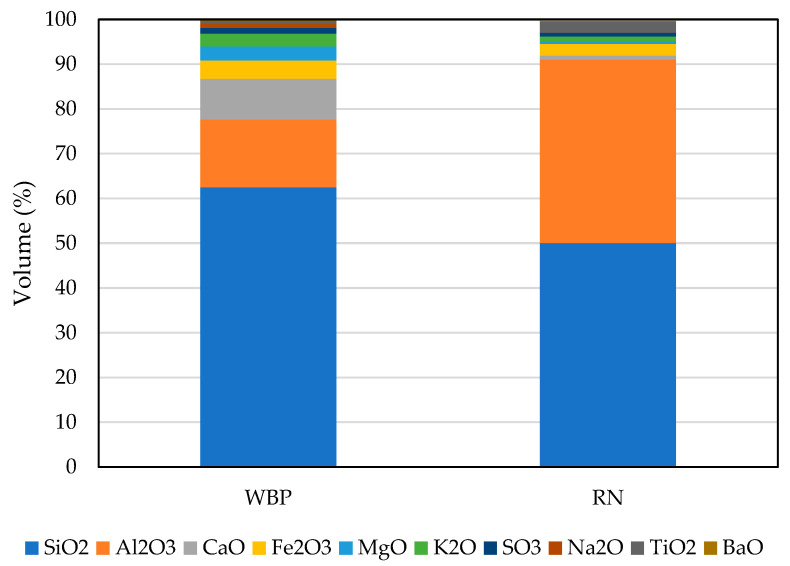
Quantified results of XRF analysis of WBP and RN.

**Figure 3:**
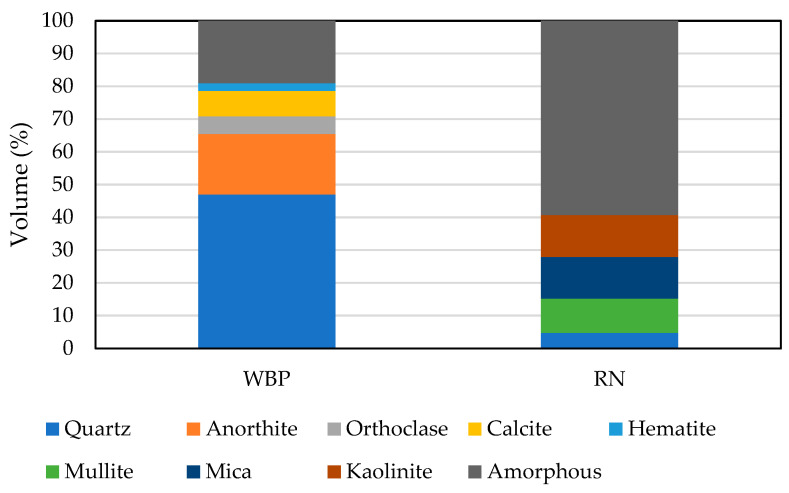
Quantified results of XRD analysis of WBP and RN.

The chemical composition of the raw materials (Figure 2) was determined by X-ray fluorescence spectroscopy to establish the oxide balance relevant for dissolution and subsequent gel formation. To assess the proportion of reactive amorphous phases versus crystalline components, quantitative phase analysis was carried out by X-ray diffraction. The diffractograms were interpreted using the HighScore Plus database, and Rietveld refinement with ZnO as an internal standard allowed for reliable quantification of amorphous content (Figure 3). This combined approach provided a consistent dataset linking particle fineness, chemical composition, and phase assemblage to the expected reactivity of each precursor in the alkaline environment. The relatively low amorphous content of WBP can be attributed to the mineral transformations that occur during high-temperature firing of clay materials and complies with previous research performed on brick-based powders [36,37,38]. At these conditions, crystalline phases such as mullite and persistent quartz become dominant, reducing the fraction of disordered aluminosilicates available for alkaline dissolution. In contrast, metakaolin originates from the dehydroxylation of kaolinite, a process that disrupts the long-range crystalline structure and produces a highly disordered network with enhanced reactivity. Lower content of the amorphous phase is also related to contamination by mortar, which increases the presence of crystalline components. RN contains significantly higher content of the amorphous phase, which predetermines its higher reactivity rate, in line with previous findings [14,39].

### 2.2. Mix Design

The selection of WBP/RN ratios was guided by preliminary trials and previous research indicating that partial replacement of WBP provides a meaningful balance between reactivity and workability. This range was therefore chosen as the most relevant for systematically assessing the synergistic effects on reaction kinetics. The solid alkaline reagents (KOH) were first dissolved in distilled water to prepare 8M potassium hydroxide, mixed with potassium silicate (M = 1.7), and consequently mixed with precursors according to the ratios listed in Table 1. The constant liquid-to-solid ratio of 0.45 was kept. The fresh pastes were poured into prismatic molds (20 × 20 × 100 mm) and allowed to harden under ambient laboratory conditions (21 °C, 50% RH) for the first 48 h. Following this initial setting stage, the specimens were transferred to a controlled chamber, where both temperature and relative humidity were regulated, and maintained under these conditions for 28 days to ensure consistent curing.

### 2.3. Used Physico-Chemical and Mechanical Assessment Methods

The evolution of setting was determined using a Vicat apparatus in compliance with EN 196-3 [40]. Fresh pastes of standard consistency were placed in molds and maintained at (20 ± 1) °C and ≥90% relative humidity. The time at which the needle ceased penetration 6 ± 3 mm above the plate was taken as the onset of rigidity, while final set was identified once no further indentation was visible, corresponding to a penetration depth ≤ 0.5 mm.

To gain deeper insight into the reaction kinetics, isothermal calorimetry was performed using a TAM Air calorimeter (TA Instruments, New Castle, DE, USA). The instrument, equipped with eight twin channels, enabled continuous monitoring of heat flow in 20 mL ampoules. Measurements were conducted in the range of 5–90 °C with a resolution of approximately 4 µW, allowing for the characterization of both rapid initial dissolution and longer-term gel formation processes.

Bulk density, skeletal density, and total open porosity were evaluated to assess the physical characteristics of the hardened composites. Bulk density was obtained from sample mass and volume, while helium pycnometry (Pycnomatic ATC, Thermo Fisher Scientific, Waltham, MA, USA) provided the skeletal density. The difference between these values was used to calculate total open porosity, giving insight into pore connectivity and packing efficiency. All reported values represent the average of three independent measurements. The standard deviation was within acceptable limits, confirming the reproducibility of the results.

Mechanical performance was assessed on prismatic specimens prepared and cured under controlled laboratory conditions. Testing was carried out using a VEB WPM Leipzig universal testing machine with a maximum load capacity of 3000 kN. Flexural strength was determined by the three-point bending method in accordance with EN 12390-5 [41]. For each mixture, three prisms with nominal dimensions of 40 × 40 × 160 mm were tested, with the load applied at a constant displacement rate until failure. Following flexural testing, each prism was split into two halves that were subsequently used for compressive strength determination, ensuring the efficient utilization of specimens and direct comparability of results. Compressive tests were performed according to EN 12390-3 [42] on six half-prisms per mixture. The load was applied perpendicularly to the casting direction at a controlled loading rate of 2.4 kN/s until failure. All values reported represent the mean of three independent measurements (flexural) or six independent measurements (compressive), with standard deviations confirming the reproducibility of the results.

Finally, pore size distribution was characterized using mercury intrusion porosimetry (MIP; Pascal 140 and Pascal 440, Thermo Fisher Scientific, Prague, Czech Republic). This method provided access to pores ranging from 3 nm to 100 µm, under pressures up to 400 MPa. Prior to intrusion, samples were evacuated under low pressure to remove residual gases, after which mercury was introduced, with a contact angle of 140°. This analysis enabled the detailed evaluation of capillary versus gel pore fractions, which are critical for understanding transport properties and strength development.

### 2.4. Life Cycle Assessment Analysis

#### 2.4.1. Goal, Scope and Functional Unit

This study quantifies the cradle-to-gate environmental footprint of alkali-activated pastes formulated from waste brick powder (WBP) and metakaolin residuum (RN) by Life Cycle Assessment (LCA) analysis. For this purpose, Simapro 8.5 and the Ecoinvent LCA database were employed. Methods follow ISO 14040/44 [43,44] and use IPCC GWP_100_ assumptions. The objective is to compare blend ratios, identify dominant contributors, especially alkali activators and precursor supply, and provide a transparent basis for benchmarking against ordinary Portland cement (OPC) paste. Results are reported per 1 kg of fresh paste.

##### System Boundary

**Figure 4:**
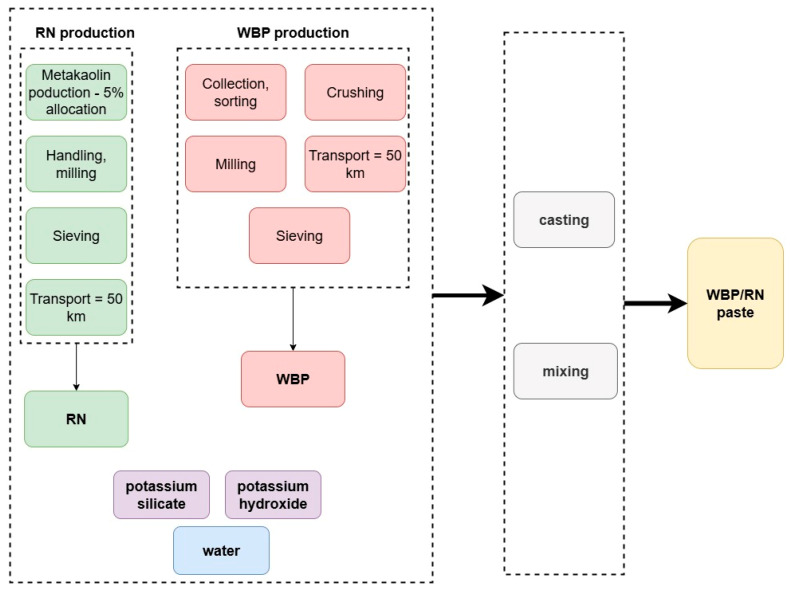
Boundary conditions.

A cradle-to-gate boundary is adopted: raw-material acquisition and processing, all transports, and paste production (mixing utilities) at the plant are included. The use-phase and end-of-life are excluded due to lack of relevant data. Considered processes, materials, and boundaries are depicted in Figure 4.

#### 2.4.2. Life-Cycle Inventory

WBP is treated as a post-consumer construction and demolition waste. The model therefore includes collection at the demolition site, road transport to the CDW facility (50 km), mechanical sorting and de-contamination, crushing, milling to the target fineness, sieving (0.125 mm mesh size), and thermal drying to the moisture specified by the mix design. Electricity for conveyors, screens, and mills; thermal energy for drying (based on moisture removed, latent heat, and dryer efficiency); dust-control fans/filters; and internal handling are inventoried per tonne of delivered powder [32]. A final transport from the CDW facility to the paste plant is included. Upstream burdens from the original brick manufacture are cut off in the attributional base case; process yields are applied so that energy and emissions are allocated to the delivered mass. RN is modeled as an industrial by-product from a metakaolin line. Following the allocation rule, RN accounts for 5% of the cradle-to-gate environmental burden of metakaolin production by economic allocation [45]. This apportions mining/beneficiation of the raw kaolin, calcination heat and power, and post-processing. Any RN-specific handling (drying, optional milling, internal transport) is added.

Potassium hydroxide is modeled via the membrane route and potassium silicate as a modulus-specified aqueous solution produced by furnace melting and dissolution; both are inventoried at delivered solids content and include upstream co-product allocation, as per the background datasets. Mixing water (municipal) and any superplasticizer are included at as-used concentrations. Site electricity follows the national or EU grid mix for the production year [46], thermal fuels for drying are modeled with measured or engineering-estimate efficiencies. All transportation to the plant is represented as tonne-kilometers by mode, using appropriate freight emission factors [47,48]. Primary data include exact mix proportions, measured energy for milling/drying/mixing, moisture contents before/after drying, and transport distances. Background data for alkaline activators solutions, electricity mixes, fuels, municipal water, generic CDW sorting/crushing, and road freight are taken from the Ecoinvent database [49,50]. A sensitivity analysis testing alternative allocation choices and transport distances to involve the regional factors was employed.

## 3. Results and Discussion

**Figure 5:**
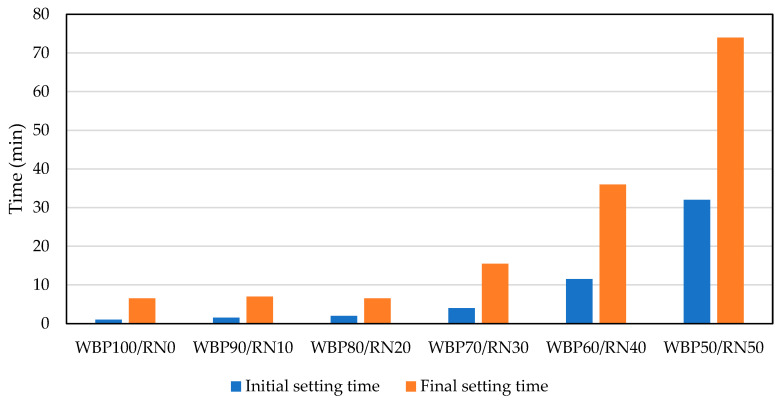
Setting characteristics of designed WBP/RN paste samples.

Figure 5 presents the initial and final setting time of alkali-activated pastes prepared with varying proportions of WBP and metakaolin residue (RN). The setting response of the WBP/RN pastes is strongly composition-dependent and evolves non-linearly as the blend moves from a WBP-dominated to an RN-dominated chemistry. As shown, WBP100/RN0 hardens almost instantaneously (initial 1.0 min; final 6.5 min). This flash behavior is consistent with the mineralogical signature of fired clay fines, whereas WBP carries Ca-crystalline phases (e.g., anorthite with minor calcite) and a very fine PSD, so in an alkaline solution it provides abundant nucleation sites for early C-(A)-S-H-type products. A rigid network percolates within minutes, even though the overall amorphous reservoir is modest. Fast early stiffening for WBP precursors has been noted for alkali-activated systems, especially when fine fractions and Ca-rich phases are present. This contrasts with the much longer induction periods typical of low-Ca, purely aluminosilicate precursors [51]. The high crystallinity of WBP limits the availability of dissolvable Si and Al, as minerals such as quartz, feldspars, and anorthite dissolve slowly at room temperature. However, CaO presence exceeding 9 wt.% was found as a factor explaining a very rapid initial hardening via early C-(A)-S-H-type precipitation, a behavior widely reported for brick-derived precursors [21]. In addition, the results of Robayo-Salazar [52] did not reveal this phenomenon, probably because of the significantly lower CaO content (0.73 wt.%). Ouda et al. [53] utilized the waste brick powder with 2.94 wt.% CaO without any adverse effects on the material performance, while the molarity of the activator played a more important role. This finding complies with Skyrianou et al. [54], who suggest the presence of CaO as an agent for a reduction in setting time. A simple indicator illustrates the imbalance in the WBP molar SiO_2_/Al_2_O_3_ ratios, 7.0 and CaO/SiO_2_ = 0.16, favoring Ca-assisted precipitation on particle surfaces but not the continuous, aluminosilicate-rich network associated with long-term strength. The application of a minor portion of RN does not immediately change this regime. Paste samples WBP90/RN10 and WBP80/RN20 kept the initial/final set in the minutes range (between 1.5 and 2.0/6.5–7.0 min). Here, RN supplies extra soluble Si and Al but, at these levels, the kinetics are still governed by WBP surface precipitation (Ca). This balance explains the marginal change in setting times. Similar behavior has been observed in studies where a reactive aluminosilicate is blended into a Ca-bearing brick-based matrix, so small additions temper the flash-set tendency without fundamentally changing the early mechanism [55].

A more significant change can be observed when moderate RN dosage is applied. At 30% RN, the initial and final set extend to 4 and 15.5 min, and for 40–50% RN they lengthen further to 11.5/36 and 32/74 min, respectively. Chemically, the blend shifts toward higher Si/Al and lower effective Ca/Si; mineralogically, the amorphous fraction rises [56]. The hardening mechanism therefore transitions from rapid Ca-triggered precipitation to geopolymeric polycondensation dominated by aluminosilicate gels, which prolong the induction period and slower network build-up at ambient temperature [57]. Calcination of kaolinite destroys long-range order and yields a highly amorphous aluminosilicate that dissolves readily, releasing Si and Al species over extended timescales. Its molar SiO_2_/Al_2_O_3_ equal to 2.1 is much closer to the range typically associated with robust N-A-S-H frameworks (Si/Al = 1.3–2.5, and the low CaO/SiO_2_ (=0.02) shift the system toward geopolymeric polycondensation rather than Ca-triggered flash precipitation [9]). Residual mullite and mica act largely as inert fillers, while any unreacted kaolinite indicates that the residue contains both highly reactive phases.

The results are consistent with studies on blended alternative precursors [25,58,59]. In alkali-activated systems where brick fines are combined with a more amorphous source (e.g., fly ash, slag, or calcined clays), modest additions of the reactive phase moderate flash setting while sustaining later gel growth; at higher replacements, setting steadily lengthens as the amorphous component controls the kinetics [60,61,62]. For instance, fly-ash/brick-powder binders and red-ceramic-waste/slag pastes show extended workable time and improved later reactivity as the amorphous share increases [55]. Such trends are attributed to a shift from Ca-assisted precipitation toward aluminosilicate polycondensation and to the different dissolution rates of the blended phases.

**Figure 6:**
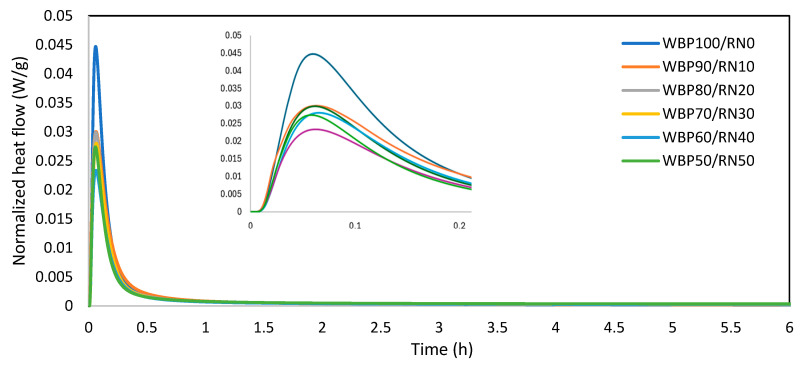
Reaction heat release of WBP/RN paste samples.

Figure 6 illustrates the reaction heat evolution of WBP/RN alkali-activated pastes. As shown, all samples exhibited an intense exothermic signal with a peak 6–7 min after mixing. This rapid heat evolution can be attributed to the initial wetting of particles and the rapid dissolution of Si and Al phases, as reported in previous research focused on the characterization of AAMs’ reaction kinetics [63,64]. The highest peaks are assigned to WBP-rich samples despite a lower amorphous content. Specifically, WBP100/RN0 reached above 0.040 W/g, almost double compared to WBP50/RN50 (0.026 W/g). When WBP was partially replaced with 10–20% RN, the maximum heat flow dropped markedly, falling into the range of 0.022–0.030 W/g. Increasing the RN content further caused a progressive decline in peak intensity, with the WBP60/RN40 mixture showing the lowest maximum value (0.02 W/g). Interestingly, this response was accompanied by a broader peak, suggesting that RN addition not only reduces the intensity of the initial reaction but also spreads the dissolution–polycondensation processes over a longer period. Similar results were noticed in previously published papers pointing out the significance of particle size on reaction heat evolution [65]. A limited reactivity of WBP particles was also noticed by Rakhimova [38], who highlighted the importance of blending brick-based precursors with fly ash of slag to attain sufficient reactivity. On the other hand, the issue of rapid setting is poorly discussed. From an application standpoint, the modulation of reaction heat by RN addition is directly relevant for controlling workability and setting in alkali-activated binders. High exothermic peaks in WBP systems cause flash setting, limiting their practical use, while the broader and attenuated signals in WBP/RN blends provide longer processing windows and more stable rheology. Such adjustment of reaction kinetics is essential for reliable casting and extrusion in construction applications.

XRD results for the reacted pastes indicate a gel-dominated composition with residual crystalline phases from the precursors (see Table 2). In WBP-rich binders, reflections of quartz and feldspathic phases (anorthite/orthoclase) persist, while approx. 45% can be assigned to an amorphous aluminosilicate gel. In line with the increase in RN, the amorphous presence intensifies. This evolution mirrors the calorimetry, with lower initial heat flow but higher cumulative heat reflecting slower, sustained dissolution of the highly disordered metakaolin residue and progressive polycondensation into N-A-S-H and mixed C-(N)-A-S-H gels. The trend agrees with current knowledge for metakaolin geopolymers, which link longer induction/acceleration periods to increased gel yield and a denser cross-linked network at later ages [66,67]. In some blends, especially at higher alkalinity or extended curing, low-intensity reflections consistent with zeolitic intermediates (e.g., hydrosodalite/analcime/faujasite) can appear atop the amorphous background [37]. Such secondary crystallization is frequently reported in aluminosilicate AAMs and is interpreted as a reorganization of the primary gel [25,68]. The extent depends on Si/Al and Na/Al ratios and the activator modulus [69]. Overall, the phase composition explains the observed reaction kinetics shown in Figure 6. WBP supplies early Ca-assisted nucleation (fast set, low cumulative heat), while RN contributes amorphous, Al-rich species that sustain gel growth (delayed set, higher cumulative heat). The resulting mixed N-A-S-H/C-(N)-A-S-H network underpins the rising strength with RN addition and the improved microstructural cohesion [70]. Similar precursor-interaction effects, where a less reactive ceramic waste is blended with a highly amorphous calcined clay to balance kinetics and maximize gel yield, have been shown to enhance strength and microstructure in brick-/metakaolin-based AAMs [71]. On the other hand, the study of Liang et al. [55] concluded that there was a positive effect of FA replacement by WBP, a higher content of the amorphous phase, which is contradictory to the presented results.

**Figure 7:**
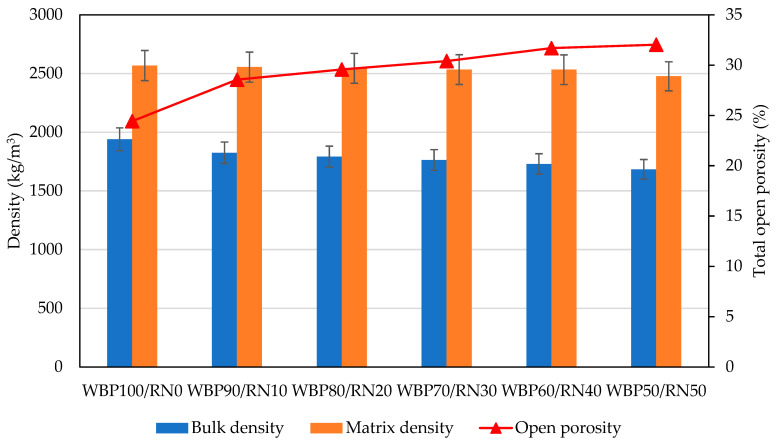
Basic material properties of WBP/RN paste samples.

**Figure 8:**
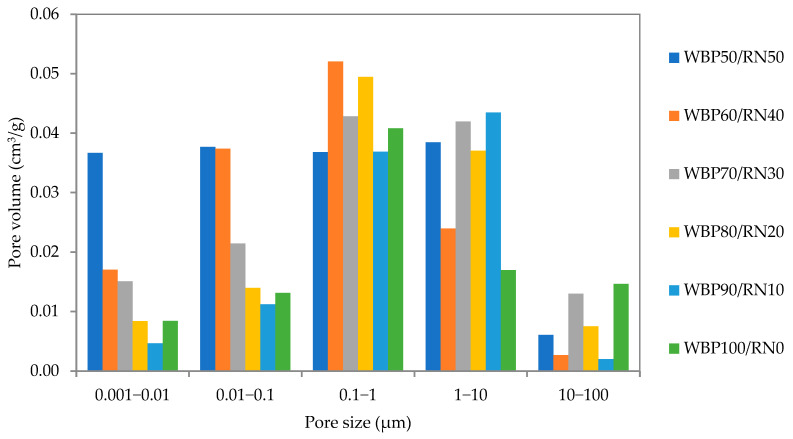
MIP analysis of WBP/RN paste samples.

The basic material characteristics of bulk, matrix density, and total open porosity are presented in Figure 7. The highest bulk density was achieved by the WBP100/RN0 sample (1940 kg m^−3^), while the porosity was about 24%. As the share of RN increased, bulk density decreased accordingly, to 1680 kg m^−3^ for WBP50/RN50, while total porosity exceeded 30%. As the powdered properties of both precursors are in the same range, the matrix density was affected only to a minor extent. A more detailed insight into the microstructure of the designed samples is provided by the performed MIP analysis, as plotted in Figure 8. The WBP100/RN0 paste contains the smallest pore volume across the tested mixtures, most notably within the 0.1–1 µm capillary range. Adding RN shifted the pore volume in the 0.1–1 µm range proportionally, which becomes the principal contributor to porosity for the 30–50% RN mixtures. The WBP50/RN50 composition achieved the largest cumulative pore volume over almost the entire size range. Importantly, lower RN dosages also induced the formation gel pores, absent in WBP100/RN0, consistent with more extensive gel formation from the amorphous-rich RN [12]. It should be acknowledged that the MIP results are subject to drying-related issues, as the removal of pore solution prior to measurement may induce shrinkage or microcracking. These effects can enlarge the measured pore volume and alter the apparent pore size distribution. However, since identical preparation procedures were applied to all mixtures, the relative trends between the samples remain comparable.

These textures can be rationalized by the previously discussed findings. A rapid setting of WBP100/RN0 caused the liquid phase to immobilize before redistribution could occur, thereby locking in the compact particle arrangement and suppressing overall porosity, and resulting in a higher bulk density [72]. As the application of RN induces prolonged plasticity, this extension allows for air migration and water movement, which favors the development of larger capillary pores and raises total porosity [22]. At the same time, the RN contributes a sustained supply of soluble Si-Al species, generating additional gel, hence the emergence of finer gel pores at moderate RN levels and the higher cumulative heat release. In a nutshell, the application of RN increases the reaction extent and gel yield but, if dosed too high without compensating mix adjustments, it also promotes capillary porosity growth [73]. The utilization of metakaolin together with ceramic waste was found to be beneficial in terms of compressive strength due to modification of the reaction kinetics and pore space formation by Statkauskas et al. [26] and Alghamdi et al. [27].

**Figure 9:**
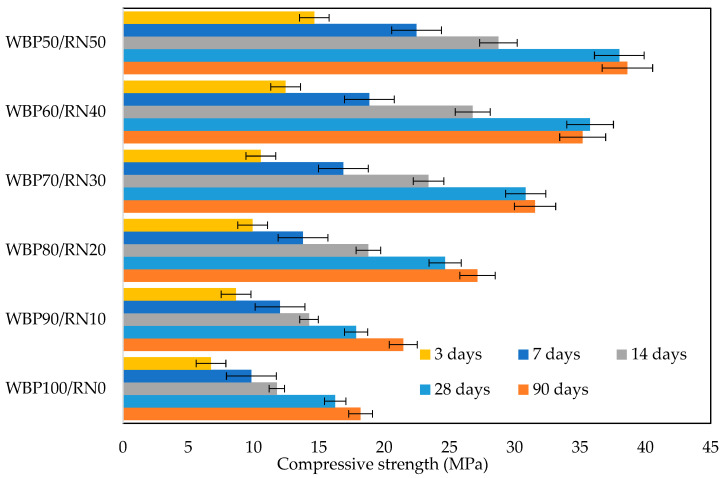
Compressive strength evolution at 3, 7, 14, 28, and 90 days.

Compressive strength increases systematically with RN content at all ages, as shown in Figure 9. From 3 to 7 days, the WBP-rich pastes have already hardened sufficiently, but the strength lags behind the RN-rich mixes by a clear margin. This early-age gap is consistent with the phase composition. Specifically, a pure WBP paste contains a low amorphous fraction and is dominated by quartz/anorthite. It sets almost instantly, yet the amount of gel that actually forms in the first hours is limited, thus the matrix “locks” around largely unreacted angular relics and stores internal flaws (microcracks at grain contacts), which affect early compressive strength [9]. Adding RN (10–30%) provides a supply of additional amorphous aluminosilicates that sustains dissolution beyond the flash-set window. Higher 3–7 d strengths than WBP100/RN0 and smoother, more continuous strength development thereafter indicate that gel formation continues after the WBP matrix has already rigidified. Between 7 and 28 days, the rate of strength increase steepens most for the 20–40% RN samples and complies with the changes observed in the distribution of the pore sizes. From 28 to 90 days, the mechanical strength increases further, but to a lesser extent. The RN-rich mixes (40–50%) show the largest late-age increment, consistent with continuing polymerization/structural rearrangement in amorphous-rich samples, in line with previous findings [73,74]. The effect on compressive behavior is still positive, as the load-bearing path benefits more from gel continuity and cross-link density than from a marginal rise in total voids. This finding is a contradiction, as the highest compressive strengths usually coincide with a lower total porosity in cement-based materials [62]. However, in AAMs, pore type and connectivity, together with mineralogical composition, play a more important role [75,76]. As summarized in Sharmin et al. [77], even more favorable findings can be identified in the available literature, but the vast majority is related to the beneficial effect of GBFS. The research performed by Riyap et al. [78] shows that even more favorable results can be achieved by mixing with metakaolin and rice husk ash, notwithstanding that the mixed design is not clearly presented. The conclusions of Ouda and Gharieb [53] demonstrate the positive role of the elevated temperature treatment to promote dissolution and strength development. A minor addition of Portland cement may significantly enhance the development of the mechanical strength and improve the application potential, as suggested by Robayo-Salazar et al. [52].

**Figure 10:**
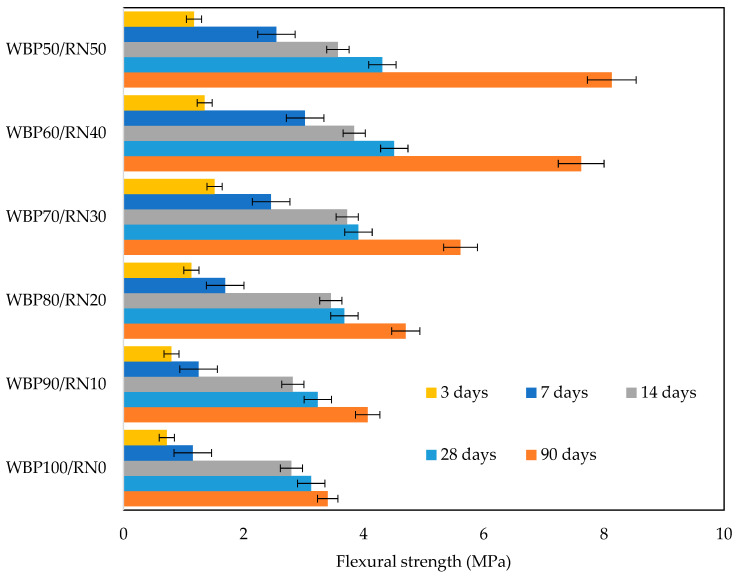
Flexural strength evolution at 3, 7, 14, 28, and 90 days.

Flexural strength (see Figure 10) develops even more sensitively to these microstructural changes. At 3–7 days, differences across mixes are modest because all matrices are still flaw-ridden and crack initiation is governed by the weakest links at unreacted grain contacts. With curing, the 20–50% RN pastes show a disproportionate rise in flexural strength, culminating around 7–8 MPa at 90 days for 40–50% RN, while WBP100/RN0 reaches values close to 3–3.5 MPa. The reason is twofold. First, the thicker reaction rims and secondary gel infill generated by RN reduce notch sensitivity. Thus, cracks are forced to propagate through a more tortuous, cohesive network rather than along sharp WBP grain boundaries. Second, introducing RN increases the proportion of short-range-ordered aluminosilicate gel, raising cross-linking and interparticle bonding. Comparable microstructure improvements are reported when moderate ceramic dosages are correlated with reactive phases. For example, MK-slag mortars with red brick strengthen as the gel becomes continuous, whereas excessive brick coarsens capillaries and suppresses gains [27]. Jurado-Contreras et al. [79] blended with biomass ash/ceramic waste, which shows slower early kinetics but strong late-age strength once the amorphous component refines the pore system. Pairing WBP with a more reactive amorphous source yields the same outcome in WBP/slag systems as reported in [51]. Relative to conventional binders, the best WBP/RN pastes in this study (≥40% RN) reach 31–36 MPa at 28 d and 35–39 MPa at 90 d in compression. Many OPC pastes can exceed these compressive values; however, considering the broad application of low-grade concrete, the benefits may arise from the significantly lower environmental footprint delivered by the utilization of waste products.

### LCA Analysis

**Figure 11:**
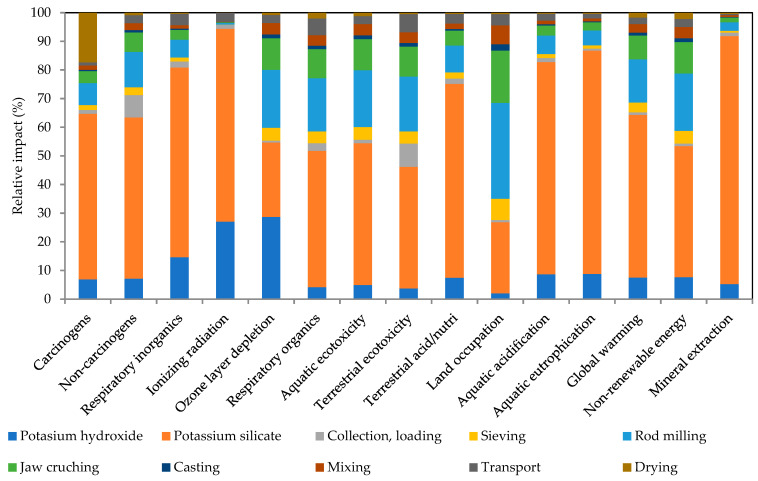
Contribution LCA analysis of WBP100/RN0.

**Figure 12:**
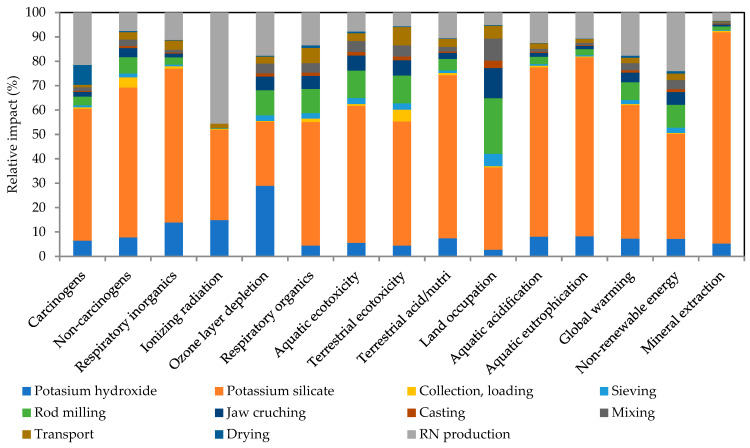
Contribution LCA analysis of WBP50/RN50.

The results of the LCA contribution analysis are presented in Figure 11 (WBP100/RN0) and Figure 12 (WBP50/RN50). Here, cradle-to-gate results show a consistent pattern across WBP/RN AAMs pastes. Specifically, the alkali activators dominate the environmental footprint, in line with previous findings [78,80,81], while the precursors are associated with notably lower impact, particularly when sourced locally (50 km considered within the analysis) and processed efficiently. In most categories, the environmental impact of alkaline solutions reached around 60%, even reaching 90% for the mineral extraction. Considering the processing steps associated with precursors, milling was identified as the most contributing processing step. As shown, the application of alkaline activators controls the environmental footprint in both formulations (55–57% from waterglass and approx. 7% from KOH), while the choice of precursor mix primarily redistributes the remaining share between RN production and the mechanical processing of WBP. This indicates that, for environmental optimization at the paste level, the most effective levers lie in the activator system (e.g., reducing silicate demand or modifying the silicate route/modulus), whereas gains from altering WBP/RN proportions will be secondary and will depend on the allocations ratio for the main product and the intensity of WBP processing and transportation distance.

**Figure 13:**
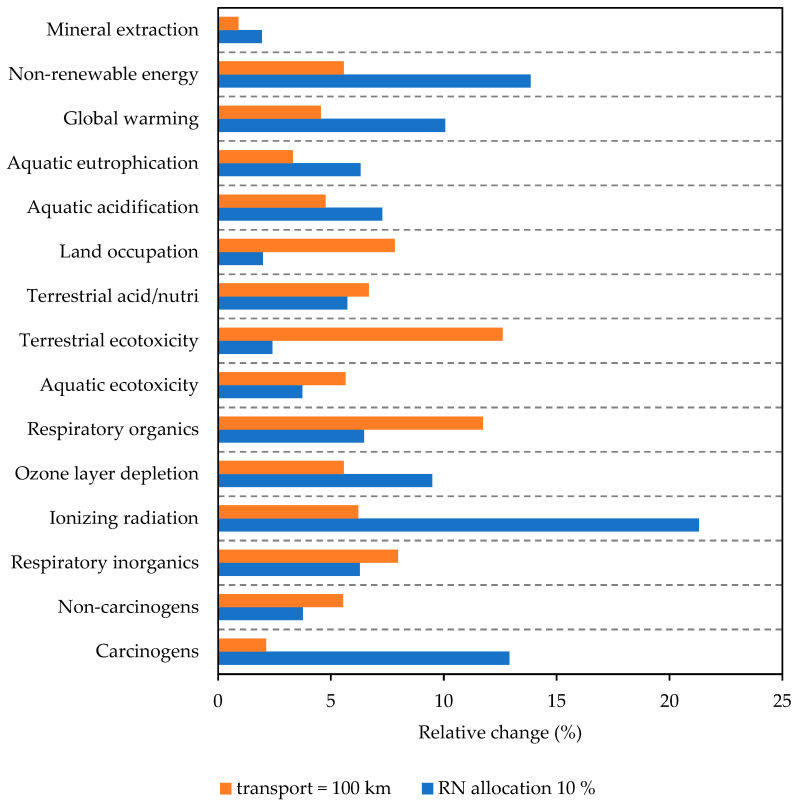
Sensitivity analysis.

To cover these variations, the sensitivity analysis was performed considering the increased transportation (100 km) and higher allocation of environmental burden associated with the RN production. The relative change in the calculated indicators is plotted in Figure 13. Here, the sensitivity analysis describes a more pronounced impact of RN allocation on the change in transport distance in categories such as ionizing radiation, carcinogens production, non-renewable energy consumption, and global warming potential. These adverse effects are related to the combustion of fossil fuels used for the calcination of metakaolin [82]. Considering the current focus on the reduction in emissions of CO_2eq_, both materials differ in global warming potential only slightly due to the same dosage of activators. The specific values for each mix are provided in Table 3. The global warming potential of the designed binders is about 40% lower compared to the results of Robayo-Salazar [52], who mixed the red brick powder with minor dosages of Portland cement to boost the mechanical performance. For better comparability with existing studies, we also report results per cubic meter; on this basis, the lower bulk density of mixes with higher RN content offsets the slightly higher CO_2_ emissions associated with RN. Specifically, moving from WBP100/RN0 to WBP50/RN50, emissions per m^3^ dropped from 408 to 393 kg CO_2eq_/m^3^. This profile matches the literature on blended waste systems (ceramic/brick with MK, FA, slag, or residues) [83]. The authors concluded that the activator impact is the principal hotspot, while considering that local CDW streams contribute little compared to the activator [84]. Studies on brick/MK(-waste), ceramic/MK, PC, and ceramic/slag systems repeatedly report the same levers as introduced here [13,24,33,35,55]. Specifically, minimized transport distances, thoughtful application of residue allocations, and rationalized activator dosage deliver meaningful GWP reductions compared to binders based on Portland cement [85,86,87,88]. The role of the optimized precursors processing, including the milling machinery and duration, also significantly affects the results [64]. The results calculated as kg CO_2e_/m^3^ show a significant reduction compared to OPC paste. Based on Cembureau data [89], CO_2e_/m^3^ for Portland cement paste corresponds to approximately 1000 kg CO_2e_/m^3^; thus, the AAM paste samples designed in this study provide about 60% savings, considering the strength level to be similar. The valorization of the waste production into a sustainable binder provides a promising future outlook meeting the ambitious targets as well as a pathway for a reduction in waste production.

## 4. Conclusions

The results of this study demonstrate that blending waste brick powder (WBP) with metakaolin residue (RN) provides a practical route to overcoming the limitations of single-precursor alkali-activated binders. The work highlights several important contributions. It establishes a direct link between reaction kinetics, mineralogical evolution, pore structure, and mechanical performance in blended WBP/RN pastes. The results underscore the potential of valorizing demolition waste and industrial residues as part of the EU circular economy, while pointing to activator optimization as the next major lever for further environmental gains. The main findings should be highlighted as follows:

- Pure WBP paste exhibited flash setting within minutes, governed by Ca-bearing crystalline phases and fine particle size, but this was accompanied by limited reactivity and modest strength gains. With increasing RN content, setting was progressively extended, the sharp calorimetric peak broadened, and reaction kinetics were sustained over longer periods. This behavior reflects the shift in mineralogy from a crystalline-rich, Ca-driven system toward one dominated by amorphous aluminosilicates, in which dissolution and gel growth proceed more gradually but more extensively.
- The evolution of phase assemblages provides a mechanistic explanation for these trends. As RN content increased, the amorphous fraction rose, while quartz and anorthite declined, leading to the formation of more continuous N-A-S-H or C-(N)-A-S-H gels. This transformation directly influenced microstructural development.
- Although total porosity increased with RN, the pore system became refined through the formation of gel-scale pores and better gel connectivity, which in turn enhanced strength. The compressive strength of RN-rich pastes reached 31–36 MPa at 28 days and up to 39 MPa at 90 days, with flexural strengths approaching 8 MPa.
- Results identify an optimal design proportion of about 30–50% RN, with Si/Al ratios around 2.0–2.5 and (Na + K)/Al close to 0.8–1.0, where setting behavior, reactivity, and strength development are well balanced. Finally, carbon footprint analysis shows that all mixes have similar cradle-to-gate impacts (392–408 kg CO_2_e/m^3^), dominated by the activator, but provide about a 60% reduction compared to Portland cement paste. Sensitivity shows RN allocation affects results more than adding 100 km transport.

Beyond laboratory performance, the valorization of WBP and RN into alkali-activated binders offers clear potential for recycling industrial residues and contributing to circular economy strategies. The moderated reaction kinetics observed in blended systems may also facilitate scale-up by improving handling during mixing and casting, which is crucial for industrial production. The role of the CaO in the WBP should be carefully considered, and CaO-rich WBP can be utilized for shortening or modulating setting time. At the same time, open challenges remain: the need to standardize processing routes, verify long-term durability under variable exposure conditions, and address the natural variability of secondary raw materials. Further research should also explore environmental assessments such as energy demand and carbon footprint, in order to quantify the sustainability benefits and define the conditions for practical implementation in the construction sector.

## Figures and Tables

**Table 1:** Mix design ratios.

Sample	WBP (%)	RN (%)	WG (Sodium Silicate) (%)	KOH 8 M (%)	Plasticizer (%)	Water (%)
WBP100/RN0	65.93	0.00	23.85	5.71	0.99	3.52
WBP90/RN10	59.34	6.59	23.85	5.71	0.99	3.52
WBP80/RN20	52.75	13.19	23.85	5.71	0.99	3.52
WBP70/RN30	46.15	19.78	23.85	5.71	0.99	3.52
WBP60/RN40	39.56	26.37	23.85	5.71	0.99	3.52
WBP50/RN50	32.97	32.97	23.85	5.71	0.99	3.52

**Table 2:** Quantified XRD analysis of WBP/RN paste samples.

	WBP100/RN0	WBP90/RN10	WBP80/RN20	WBP70/RN30	WBP60/RN40	WBP50/RN50
Amorphous	46.4	46.9	48.4	48.5	56.2	55.3
Quartz	31.5	23.6	24	22.4	18.8	14.3
Anorthite	15.2	12	9	10.2	5.1	6.9
Orthoclase	3	3.8	3.9	3.6	2.8	3.1
Calcite	3.9	5.3	4.7	3.5	2.8	3.2
Kaolinite	0	3.5	5.3	6.3	7.6	11.1
Mica	0	4.9	4.7	5.5	6.7	6.1
Total	100	100	100	100	100	100

**Table 3:** Results of LCA analysis.

Impact Category	WBP50/RN50	BP60/RN40	WBP70/RN30	WBP80/RN20	WBP90/RN10	WBP100/RN0
Carcinogens	2.306	2.276	2.245	2.215	2.185	2.155
Non-carcinogens	4.163	4.239	4.314	4.389	4.465	4.540
Respiratory inorganics	0.194	0.192	0.190	0.188	0.186	0.184
Ionizing radiation	1490	1356	1223	1089	955	821
Ozone layer depletion	3.07 × 10^−5^	3.08 × 10^−5^	3.08 × 10^−5^	3.09 × 10^−5^	3.10 × 10^−5^	3.10 × 10^−5^
Respiratory organics	0.0461	0.0467	0.0473	0.0479	0.0485	0.0491
Aquatic ecotoxicity	18,187	18,676	19,164	19,652	20,141	20,629
Terrestrial ecotoxicity	6437	6691	6946	7200	7454	7708
Terrestrial acid/nutri	3.580	3.572	3.563	3.555	3.546	3.537
Land occupation	7.739	8.296	8.853	9.409	9.966	10.522
Aquatic acidification	0.986	0.973	0.961	0.948	0.936	0.923
Aquatic eutrophication	0.0517	0.0511	0.0505	0.0499	0.0493	0.0487
Global warming	233.3	228.8	224.2	219.6	215.1	210.4
Non-renewable energy	3015	2979	2943	2907	2871	2835
Mineral extraction	14.57	14.56	14.56	14.56	14.56	14.57

## Data Availability

Data will be available upon request.

## References

[1] Barbhuiya S., Kanavaris F., Das B., Idrees M. (2024). Decarbonising Cement and Concrete Production: Strategies, Challenges and Pathways for Sustainable Development. J. Build. Eng..

[2] Li Q., Li Q., Wang F., Xu N., Wang Y., Bai B. (2025). Settling Behavior and Mechanism Analysis of Kaolinite as a Fracture Proppant of Hydrocarbon Reservoirs in CO_2_ Fracturing Fluid. Colloids Surf. A Physicochem. Eng. Asp..

[3] Li Q., Han Y., Liu X., Ansari U., Cheng Y., Yan C. (2022). Hydrate as a By-Product in CO_2_ Leakage during the Long-Term Sub-Seabed Sequestration and Its Role in Preventing Further Leakage. Environ. Sci. Pollut. Res..

[4] Di Filippo J., Karpman J., DeShazo J.R. (2019). The Impacts of Policies to Reduce CO_2_ Emissions within the Concrete Supply Chain. Cem. Concr. Compos..

[5] Brito J., Kurda R. (2020). The Past and Future of Sustainable Concrete: A Critical Review and New Strategies on Cement-Based Materials. J. Clean. Prod..

[6] Duxson P., Fernández-Jiménez A., Provis J.L., Lukey G.C., Palomo A., van Deventer J.S.J. (2007). Geopolymer Technology: The Current State of the Art. J. Mater. Sci..

[7] Zuo Y., Chen Y., Liu C., Gan Y., Göbel L., Ye G., Provis J.L. (2025). Modeling and Simulation of Alkali-Activated Materials (AAMs): A Critical Review. Cem. Concr. Res..

[8] Ślosarczyk A., Fořt J., Klapiszewska I., Thomas M., Klapiszewski Ł., Černý R. (2023). A Literature Review of the Latest Trends and Perspectives Regarding Alkali-Activated Materials in Terms of Sustainable Development. J. Mater. Res. Technol..

[9] Provis J.L. (2018). Alkali-Activated Materials. Cem. Concr. Res..

[10] Nehdi M., Marani A., Zhang L. (2024). Is Net-Zero Feasible: Systematic Review of Cement and Concrete Decarbonization Technologies. Renew. Sustain. Energy Rev..

[11] Filipović S., Lior N., Radovanović M. (2022). The Green Deal—Just Transition and Sustainable Development Goals Nexus. Renew. Sustain. Energy Rev..

[12] El-Naggar M.R., El-Dessouky M.I. (2017). Re-Use of Waste Glass in Improving Properties of Metakaolin-Based Geopolymers: Mechanical and Microstructure Examinations. Constr. Build. Mater..

[13] Mellado A., Catalán C., Bouzón N., Borrachero M.V., Monzó J.M., Payá J. (2014). Carbon Footprint of Geopolymeric Mortar: Study of the Contribution of the Alkaline Activating Solution and Assessment of an Alternative Route. RSC Adv..

[14] Bai C., Zheng K., Sun F., Wang X., Zhang L., Zheng T., Colombo P., Wang B. (2024). A Review on Metakaolin-Based Porous Geopolymers. Appl. Clay Sci..

[15] Gonçalves D.K.C., Lana S.L.B., Sales R.B.C., Aguilar M.T.P. (2022). Study of Metakaolins with Different Amorphities and Particle Sizes Activated by KOH and K_2_SiO_3_. Case Stud. Constr. Mater..

[16] Habert G., Ouellet-Plamondon C. (2016). Recent Update on the Environmental Impact of Geopolymers. RILEM Tech. Lett..

[17] Liu J., Doh J.H., Dinh H.L., Ong D.E.L., Zi G., You I. (2022). Effect of Si/Al Molar Ratio on the Strength Behavior of Geopolymer Derived from Various Industrial Waste: A Current State of the Art Review. Constr. Build. Mater..

[18] Mahmoodi O., Siad H., Lachemi M., Şahmaran M. (2024). Combined Application of CDWs as Precursors and Aggregates in Geopolymer Composites: A Comprehensive Rheological Analysis. J. Build. Eng..

[19] Alzeebaree R., Mawlod A.O., Mohammedameen A., Niş A. (2021). Using of Recycled Clay Brick/Fine Soil to Produce Sodium Hydroxide Alkali Activated Mortars. Adv. Struct. Eng..

[20] Robayo R.A., Mulford A., Munera J., Mejía de Gutiérrez R. (2016). Alternative Cements Based on Alkali-Activated Red Clay Brick Waste. Constr. Build. Mater..

[21] Reig L., Tashima M.M., Borrachero M.V., Monzó J., Cheeseman C.R., Payá J. (2013). Properties and Microstructure of Alkali-Activated Red Clay Brick Waste. Constr. Build. Mater..

[22] Shen J., Li Y., Lin H., Lv J., Feng S., Ci J. (2022). Early Properties and Chemical Structure Analysis of Alkali-Activated Brick Geopolymer with Varied Alkali Dosage. J. Build. Eng..

[23] Sharmin S., Sarker P.K., Biswas W.K., Abousnina R.M., Javed U. (2024). Characterization of Waste Clay Brick Powder and Its Effect on the Mechanical Properties and Microstructure of Geopolymer Mortar. Constr. Build. Mater..

[24] Cardoza A., Colorado H.A. (2024). Alkaline Activation of Brick Waste with Partial Addition of Ordinary Portland Cement (OPC) for Reducing Brick Industry Pollution and Developing a Feasible and Competitive Construction Material. Open Ceram..

[25] Hassan H., El-Gamal S.M.A., Shehab M.S.H., Mohsen A. (2023). Development of Green Ternary-Blended-Geopolymers for Multifunctional Engineering Applications. Constr. Build. Mater..

[26] Statkauskas M., Vaičiukynienė D., Grinys A., Paul Borg R. (2023). Mechanical Properties and Microstructure of Ternary Alkali Activated System: Red Brick Waste, Metakaolin and Phosphogypsum. Constr. Build. Mater..

[27] Alghamdi H., Abadel A.A., Khawaji M., Alamri M., Alabdulkarim A. (2023). Strength Performance and Microstructures of Alkali-Activated Metakaolin and GGBFS-Based Mortars: Role of Waste Red Brick Powder Incorporation. Minerals.

[28] Jin P., Li L., Li Z., Du W., Khan M., Li Z. (2024). Using Recycled Brick Powder in Slag Based Geopolymer Foam Cured at Ambient Temperature: Strength, Thermal Stability and Microstructure. Constr. Build. Mater..

[29] Valente M., Sambucci M., Chougan M., Ghaffar S.H. (2022). Reducing the Emission of Climate-Altering Substances in Cementitious Materials: A Comparison between Alkali-Activated Materials and Portland Cement-Based Composites Incorporating Recycled Tire Rubber. J. Clean. Prod..

[30] Kravchenko E., Lazorenko G., Jiang X., Leng Z. (2024). Alkali-Activated Materials Made of Construction and Demolition Waste as Precursors: A Review. Sustain. Mater. Technol..

[31] Alkhawaldeh A.A., Judah H.I., Shammout D.Z., Almomani O.A., Alkhawaldeh M.A. (2024). Sustainability Evaluation and Life Cycle Assessment of Concretes Including Pozzolanic By-Products and Alkali-Activated Binders. Results Eng..

[32] Fořt J., Černý R. (2020). Transition to Circular Economy in the Construction Industry: Environmental Aspects of Waste Brick Recycling Scenarios. Waste Manag..

[33] Wang F., Zhai J., Kan E., Norkulov B., Ding Y., Yu J., Yu K. (2024). Value-Added Recycling of Waste Brick Powder and Waste Sand to Develop Eco-Friendly Engineered Geopolymer Composite. Case Stud. Constr. Mater..

[34] Roy A., Sadiqul Islam G.M. (2024). Geopolymer Using Different Size Fractions of Recycled Brick-Based Mixed Demolition Waste. Clean. Mater..

[35] Maaze M.R., Shrivastava S. (2024). Development and Performance Evaluation of Recycled Brick Waste-Based Geopolymer Brick for Improved Physcio-Mechanical, Brick-Bond and Durability Properties. J. Build. Eng..

[36] Al-Noaimat Y.A., Chougan M., Al-kheetan M.J., Yio M.H.N., Wong H.S., Ghaffar S.H. (2023). Upcycling End-of-Life Bricks in High-Performance One-Part Alkali-Activated Materials. Dev. Built Environ..

[37] Migunthanna J., Rajeev P., Sanjayan J. (2021). Investigation of Waste Clay Brick as Partial Replacement of Geopolymer Binders for Rigid Pavement Application. Constr. Build. Mater..

[38] Rakhimova N.R., Rakhimov R.Z. (2015). Alkali-Activated Cements and Mortars Based on Blast Furnace Slag and Red Clay Brick Waste. Mater. Des..

[39] Peys A., Rahier H., Pontikes Y. (2016). Potassium-Rich Biomass Ashes as Activators in Metakaolin-Based Inorganic Polymers. Appl. Clay Sci..

[40] (2017). Methods of Testing Cement—Part 3: Determination of Setting Times and Volumetric Stability.

[41] (2007). Testing of Hardened Concrete—Part 5: Flexural Strength.

[42] (2007). Testing of Hardened Concrete—Part 5: Compressive Strength.

[43] (2006). Environmental Management—Life Cycle Assessment—Principles and Framework.

[44] (2006). Environmental Management—Life Cycle Assessment—Requirements and Guidelines.

[45] Visintin P., Xie T., Bennett B. (2020). A Large-Scale Life-Cycle Assessment of Recycled Aggregate Concrete: The Influence of Functional Unit, Emissions Allocation and Carbon Dioxide Uptake. J. Clean. Prod..

[46] Moro A., Lonza L. (2018). Electricity Carbon Intensity in European Member States: Impacts on GHG Emissions of Electric Vehicles. Transp. Res. D Transp. Environ..

[47] Zhang Y., Gong H., Jiang X., Lv X., Xiao R., Huang B. (2021). Environmental Impact Assessment of Pavement Road Bases with Reuse and Recycling Strategies: A Comparative Study on Geopolymer Stabilized Macadam and Conventional Alternatives. Transp. Res. D Transp. Environ..

[48] Fořt J., Mildner M., Černý R. (2024). Consequences of Omitting Some Important Factors in the Environmental Analyses of Commercial Sodium Silicate/Sodium Hydroxide Use for Alkaline Activation in the Light of Comparison with Cement-Based Composites. Sci. Total Environ..

[49] Wernet G., Bauer C., Steubing B., Reinhard J., Moreno-Ruiz E., Weidema B. (2016). The Ecoinvent Database Version 3 (Part I): Overview and Methodology. Int. J. Life Cycle Assess..

[50] Bulle C., Margni M., Patouillard L., Boulay A.-M., Bourgault G., De Bruille V., Cao V., Hauschild M., Henderson A., Humbert S. (2019). IMPACT World+: A Globally Regionalized Life Cycle Impact Assessment Method. Int. J. Life Cycle Assess..

[51] Fořt J., Mildner M., Keppert M., Pommer V., Černý R. (2023). Experimental and Environmental Analysis of High-Strength Geopolymer Based on Waste Bricks and Blast Furnace Slag. Polymers.

[52] Robayo-Salazar R.A., Mejía-Arcila J.M., Mejía de Gutiérrez R. (2017). Eco-Efficient Alkali-Activated Cement Based on Red Clay Brick Wastes Suitable for the Manufacturing of Building Materials. J. Clean. Prod..

[53] Ouda A.S., Gharieb M. (2020). Development the Properties of Brick Geopolymer Pastes Using Concrete Waste Incorporating Dolomite Aggregate. J. Build. Eng..

[54] Skyrianou I., Koutas L.N., Papakonstantinou C.G. (2025). Metakaolin-Based Geopolymer Mortars: Influence of Mix Design on Mechanical Properties and Durability. Constr. Build. Mater..

[55] Liang G., Luo L., Yao W. (2022). Reusing Waste Red Brick Powder as Partial Mineral Precursor in Eco-Friendly Binders: Reaction Kinetics, Microstructure and Life-Cycle Assessment. Resour. Conserv. Recycl..

[56] Mahmoodi O., Siad H., Lachemi M., Dadsetan S., Sahmaran M. (2020). Optimization of Brick Waste-Based Geopolymer Binders at Ambient Temperature and Pre-Targeted Chemical Parameters. J. Clean. Prod..

[57] Arachchige R.M., Olek J., Rajabipour F., Peethamparan S. (2023). Non-Traditional Aluminosilicate Based Alkali-Activated Mortars—Statistical Optimization of Solution Parameters and Processing Conditions for Optimal Compressive Strength, Workability and Setting Time. Constr. Build. Mater..

[58] Gharieb M., Mosleh Y.A., Rashad A.M. (2021). Properties and Corrosion Behaviour of Applicable Binary and Ternary Geopolymer Blends. Int. J. Sustain. Eng..

[59] Samarakoon M.H., Ranjith P.G., Duan W.H., De Silva V.R.S. (2020). Properties of One-Part Fly Ash/Slag-Based Binders Activated by Thermally-Treated Waste Glass/NaOH Blends: A Comparative Study. Cem. Concr. Compos..

[60] Gao X., Yu Q.L., Lazaro A., Brouwers H.J.H. (2017). Investigation on a Green Olivine Nano-Silica Source Based Activator in Alkali Activated Slag-Fly Ash Blends: Reaction Kinetics, Gel Structure and Carbon Footprint. Cem. Concr. Res..

[61] Marathe S., Sadowski Ł., Shree N. (2024). Geopolymer and Alkali-Activated Permeable Concrete Pavements: Bibliometrics and Systematic Current State of the Art Review, Applications, and Perspectives. Constr. Build. Mater..

[62] Fořt J., Afolayan A., Medveď I., Scheinherrová L., Černý R. (2024). A Review of the Role of Lightweight Aggregates in the Development of Mechanical Strength of Concrete. J. Build. Eng..

[63] Tan J., Cizer Ö., De Vlieger J., Dan H.-C., Li J. (2022). Impacts of Milling Duration on Construction and Demolition Waste (CDW) Based Precursor and Resulting Geopolymer: Reactivity, Geopolymerization and Sustainability. Resour. Conserv. Recycl..

[64] Fořt J., Vejmelková E., Keppert M., Rovnaníková P., Bezdička P., Černý R. (2020). Alkaline Activation of Low-Reactivity Ceramics: Peculiarities Induced by the Precursors’ Dual Character. Cem. Concr. Compos..

[65] Pommer V., Vejmelková E., Černý R., Keppert M. (2021). Alkali-Activated Waste Ceramics: Importance of Precursor Particle Size Distribution. Ceram. Int..

[66] Kamseu E., Beleuk à Moungam L.M., Cannio M., Billong N., Chaysuwan D., Melo U.C., Leonelli C. (2017). Substitution of Sodium Silicate with Rice Husk Ash-NaOH Solution in Metakaolin Based Geopolymer Cement Concerning Reduction in Global Warming. J. Clean. Prod..

[67] Tchakouté H.K., Rüscher C.H., Kong S., Kamseu E., Leonelli C. (2016). Geopolymer Binders from Metakaolin Using Sodium Waterglass from Waste Glass and Rice Husk Ash as Alternative Activators: A Comparative Study. Constr. Build. Mater..

[68] Chelluri S.K., Hossiney N. (2024). Performance Evaluation of Ternary Blended Geopolymer Binders Comprising of Slag, Fly Ash and Brick Kiln Rice Husk Ash. Case Stud. Constr. Mater..

[69] An Q., Pan H., Zhao Q., Wang D. (2022). Strength Development and Microstructure of Sustainable Geopolymers Made from Alkali-Activated Ground Granulated Blast-Furnace Slag, Calcium Carbide Residue, and Red Mud. Constr. Build. Mater..

[70] Guo S., Wu Y., Jia Z., Qi X., Wang W. (2023). Sodium-Based Activators in Alkali- Activated Materials: Classification and Comparison. J. Build. Eng..

[71] Vo D.-H., Hwang C.-L., Thi K.-D.T., Yehualaw M., Liao M.-C., Chao Y.-F. (2020). HPC Produced with CDW as a Partial Replacement for Fine and Coarse Aggregates Using the Densified Mixture Design Algorithm (DMDA) Method: Mechanical Properties and Stability in Development. Constr. Build. Mater..

[72] Font A., Soriano L., de Moraes Pinheiro S.M., Tashima M.M., Monzó J., Borrachero M.V., Payá J. (2020). Design and Properties of 100% Waste-Based Ternary Alkali-Activated Mortars: Blast Furnace Slag, Olive-Stone Biomass Ash and Rice Husk Ash. J. Clean. Prod..

[73] Reig L., Soriano L., Tashima M.M., Borrachero M.V., Monzó J., Payá J. (2018). Influence of Calcium Additions on the Compressive Strength and Microstructure of Alkali-Activated Ceramic Sanitary-Ware. J. Am. Ceram. Soc..

[74] Yang T., Zhu H., Zhang Z. (2017). Influence of Fly Ash on the Pore Structure and Shrinkage Characteristics of Metakaolin-Based Geopolymer Pastes and Mortars. Constr. Build. Mater..

[75] Liao G., Noguchi T. (2025). Effect of CaO-Al2O3-SiO2 Molar Ratio on Compressive Strength, Reaction Products, and Strength Prediction Model of CaO-Activated Materials. Case Stud. Constr. Mater..

[76] Seki Y., Shibayama A., Nishiyama M., Kikuchi M. (2024). Machine Learning Models for Predicting the Compressive Strengths of Ordinary Portland Cement Concrete and Alkali-Activated Materials. Sustain. Mater. Technol..

[77] Sharmin S., Biswas W.K., Sarker P.K. (2024). Exploring the Potential of Using Waste Clay Brick Powder in Geopolymer Applications: A Comprehensive Review. Buildings.

[78] Riyap H.I., Banenzoué C., Tchakouté H.K., Nanseu C.N.P., Rüscher C.H. (2021). A Comparative Study of the Compressive Strengths and Microstructural Properties of Geopolymer Cements from Metakaolin and Waste Fired Brick as Aluminosilicate Sources. J. Korean Ceram. Soc..

[79] Jurado-Contreras S., Bonet-Martínez E., Sánchez-Soto P.J., Gencel O., Eliche-Quesada D. (2022). Synthesis and Characterization of Alkali-Activated Materials Containing Biomass Fly Ash and Metakaolin: Effect of the Soluble Salt Content of the Residue. Arch. Civ. Mech. Eng..

[80] Fořt J., Mildner M., Keppert M., Abed M., Černý R. (2023). Potential of Industrial Waste as Alternative Alkaline Activator for Development of Eco-Efficient Mortars. Case Stud. Constr. Mater..

[81] Fořt J., Mildner M., Afolayan A., Hotěk P., Tang L., Keppert M., Slosarczyk A., Klapiszewska I., Klapiszewski Ł., Černý R. (2025). Alternative Binder Systems: Cumulative Assessment of Environmental and Functional Parameters. Environ. Impact Assess. Rev..

[82] Heath A., Paine K., McManus M. (2014). Minimising the Global Warming Potential of Clay Based Geopolymers. J. Clean. Prod..

[83] Li Y., Shen J., Lin H., Lv J., Feng S., Ci J. (2022). Properties and Environmental Assessment of Eco-Friendly Brick Powder Geopolymer Binders with Varied Alkali Dosage. J. Build. Eng..

[84] Bianco I., Ap Dafydd Tomos B., Vinai R. (2021). Analysis of the Environmental Impacts of Alkali-Activated Concrete Produced with Waste Glass-Derived Silicate Activator—A LCA Study. J. Clean. Prod..

[85] Ramagiri K.K., Kar A. (2021). Environmental Impact Assessment of Alkali-Activated Mortar with Waste Precursors and Activators. J. Build. Eng..

[86] Fernando S., Gunasekara C., Law D.W., Nasvi M.C.M., Setunge S., Dissanayake R., Robert D. (2022). Environmental Evaluation and Economic Analysis of Fly Ash-Rice Husk Ash Blended Alkali-Activated Bricks. Environ. Impact Assess. Rev..

[87] Abdulkareem M., Havukainen J., Nuortila-Jokinen J., Horttanainen M. (2021). Environmental and Economic Perspective of Waste-Derived Activators on Alkali-Activated Mortars. J. Clean. Prod..

[88] Ameri F., Zareei S.A., Behforouz B. (2020). Zero-Cement vs. Cementitious Mortars: An Experimental Comparative Study on Engineering and Environmental Properties. J. Build. Eng..

[89] Cao Z., Shen L., Løvik A.N., Müller D.B., Liu G. (2017). Elaborating the History of Our Cementing Societies: An in-Use Stock Perspective. Environ. Sci. Technol..

